# *Nostoc commune* Extract Attenuates Oxidative Stress and Neuroinflammation in Ischemic Optic Neuropathy Through PI3K/AKT/mTOR Signaling

**DOI:** 10.3390/antiox15050541

**Published:** 2026-04-24

**Authors:** Jia-Ying Chien, Wei-Hsun Chan, Mei-Ling Peng, Siu-Fung Chau, Hsien-Yang Tsai, Shi-Huang Lee, Yu-Chen Chen, Wai-Man Cheang, Shun-Ping Huang

**Affiliations:** 1 Department of Biochemical Science and Technology, National Chiayi University, Chiayi City 600355, Taiwan; jychien416@gmail.com (J.-Y.C.); qaz2361778@gmail.com (W.-H.C.); 2 Department of Ophthalmology, Taichung Tzu Chi Hospital, Taichung 427213, Taiwan; pml02046@gmail.com (M.-L.P.); chausiufung01@gmail.com (S.-F.C.); choihinyeung@tzuchi.com.tw (H.-Y.T.); shami@chantoil.com.tw (S.-H.L.); moonimi0219@yahoo.com.tw (Y.-C.C.); adycheang@gmail.com (W.-M.C.)

**Keywords:** non-arteritic anterior ischemic optic neuropathy, retinal ganglion cells, *Nostoc commune*, visual evoked potential, oxidative stress, PI3K/AKT/mTOR/p70S6K, neuroinflammation, cyanobacteria, neuroprotection

## Abstract

Non-arteritic anterior ischemic optic neuropathy (NAION) is a leading cause of sudden vision loss, yet no effective therapy exists to preserve retinal ganglion cells (RGCs) after ischemic injury. *Nostoc commune* (NC), an edible cyanobacterium with established antioxidant and anti-inflammatory activities, has emerged as a potential functional bioresource with relevance to ocular health. Here, we investigated the therapeutic effects of a crude aqueous extract of NC using a rodent model of anterior ischemic optic neuropathy (rAION). NC treatment significantly improved RGC survival, reduced apoptosis, attenuated macrophage and microglial activation (ED-1, Iba1), suppressed proinflammatory cytokine expression (IL-6), enhanced the reparative marker Ym1+2, and preserved optic-nerve myelination. Functionally, NC administration restored visual signaling as demonstrated by improved Flash Visual Evoked Potential amplitudes. Immunoblot analysis showed increased phosphorylation of PI3K/AKT/mTOR/p70S6K signaling components in retinal tissue following NC treatment. Proteomic profiling further demonstrated that NC extract comprises a coordinated repertoire of phycobiliproteins, antioxidant enzymes, and stress-response proteins that may collectively contribute to its biological effects. Together, these findings suggest that *Nostoc commune* extract may serve as a promising functional food-derived candidate for protecting RGCs and preserving visual function following ischemic optic neuropathy. Further studies are required to identify its active constituents, optimize formulation strategies, and evaluate its translational potential.

## 1. Introduction

Retinal Ganglion Cells (RGCs) serve as the retina’s output neurons, processing and transmitting visual signals to the brain. Their degeneration results in a profound impact on vision, leading to irreversible blindness worldwide [1]. Effective treatments are currently lacking for many conditions linked to RGC degeneration, including glaucoma, hereditary and ischemic optic neuropathies, and demyelinating diseases. A common pathological hallmark across various optic neuropathies is the apoptosis of RGCs and resultant axonal damage. Specifically, major degenerative causes, such as glaucoma and optic neuritis, involve the dysfunction or degeneration of the optic nerve axons, which inevitably progress to optic nerve atrophy [2]. Non-arteritic anterior ischemic optic neuropathy (NAION) represents the most common form of ischemic optic neuropathy, primarily damaging optic nerve axons and resulting in the death of Retinal Ganglion Cells (RGCs) within the retina. Pathologically, NAION is characterized by optic nerve swelling, axonal degeneration, RGC apoptosis, and pervasive inflammation throughout both the retina and the optic nerve. This disease primarily affects individuals over 55 years old [3,4]. Nevertheless, the pathophysiology of NAION is not fully elucidated, and no established treatments are currently available. Previous studies have identified that individuals with diabetes mellitus, hypercholesterolemia, hypertension, and obstructive sleep apnea have a higher risk of developing NAION [5,6,7]. In clinical practice, corticosteroids are the primary treatment for NAION. Unfortunately, corticosteroid treatment is not effective for all patients [8]. Currently, no widely accepted treatment or preventive method for NAION exists. The primary treatment or prevention strategy for managing non-arteritic ischemic optic neuropathy (NAION) is to address the symptoms and treat or minimize any underlying causes and contributing factors.

Evidence from both clinical patients and rodent models of NAION consistently demonstrates key pathological events, notably the infiltration of macrophages and the subsequent breakdown of the retinal–blood barrier [9]. Resting microglia continuously monitor and maintain the CNS microenvironment. During ischemic insults or CNS damage, they transition into an activated state [10,11]. In this activated state, termed the M1 or proinflammatory phase, microglia release a range of proinflammatory mediators, including interleukin-1 (IL-1), interleukin-6 (IL-6), and nitric oxide (NO). These factors not only contribute to oxidative stress but also promote the activation of NF-KB and tumor necrosis factor-alpha (TNF-α). Collectively, these cytokines and mediators enhance the recruitment of immune factors and facilitate the infiltration of peripheral immune cells, ultimately leading to neuronal damage and cell apoptosis [12,13,14]. Additionally, microglia play a key regulatory role in neuroinflammation by inducing a proinflammatory state and producing various factors that can impact neuronal survival [15]. Taken together, modulating activated microglia could be a potential therapeutic approach for ischemic optic neuropathy.

*Nostoc commune* (NC) is a cyanobacterium, also known as a blue-green alga, that is widely distributed across both aquatic and terrestrial environments worldwide [16]. NC has been extensively reported to harbor diverse and potent bioactive properties, including antioxidant, anti-inflammatory, antitumor, antilipidemic, and anti-aging effects. Historically, Nostoc species have maintained their popularity in both gourmet cuisine and herbal medicine. Consequently, for decades, researchers have actively investigated the utility of *Nostoc commune* in various medical, cosmeceutical, and nutraceutical applications [17,18,19]. A previous study demonstrated that *Nostoc commune* can modulate neuroinflammation and oxidative stress by regulating the expression of interleukin-6 (IL-6) and tumor necrosis factor-alpha (TNF-α) in a mouse model of schizophrenia (SCZ) [20]. Given these findings, the development of NC, particularly by focusing on its anti-inflammatory and antioxidative effects through the modulation of molecular signaling pathways, presents a promising avenue for NAION therapy.

To investigate the pathological mechanisms of ischemic optic neuropathy, we employed a rodent anterior ischemic optic neuropathy (rAION) model that reproduces key features of the disease [9,21,22]. We further hypothesized that NC extract may alleviate visual dysfunction by modulating ischemia-related inflammatory and neurodegenerative processes.

## 2. Materials and Methods

### 2.1. Animals

Male Wistar rats (4 weeks old, 75–100 g) were utilized in this study. Animals were sourced from BioLASCO Taiwan Co., Ltd. (Taipei, Taiwan). They were maintained under controlled environmental conditions with regulated temperature and a 12 h light/dark cycle, with free access to standard chow and water. All procedures were conducted in accordance with the ARVO Statement for the Use of Animals in Ophthalmic and Vision Research and were approved by the Institutional Animal Care and Use Committee of National Chiayi University (IACUC No. 113041). For all experimental procedures, anesthesia was administered via intramuscular injection of ketamine (100 mg/kg) combined with xylazine (10 mg/kg). Mydriasis and topical anesthesia were induced using 0.5% tropicamide and 0.5% proparacaine hydrochloride (Alcaine^®^, Alcon, Mechelen, Belgium), respectively. Each experimental group included six animals (*n* = 6).

### 2.2. NC Extract Preparation and Administration

*Nostoc commune* (NC) was kindly provided by Dr. Wei-Chung Liu and Dr. Nien-Tzu Keng at Tzu Chi University, Hualien, Taiwan (23.997476, 121.564972). After removal of visible debris, NC biomass was rinsed three times with sterile deionized water and drained on sterile gauze. The cleaned biomass was homogenized with deionized water at a ratio of 1:2 (*w*/*v*; g wet of NC biomass: water) using a high-speed blender until a uniform slurry was obtained. The homogenate was subsequently subjected to a high-pressure cell disruptor (CF1 Cell Disruptor, Constant Systems, Daventry, United Kingdom) at 20 kpsi (~1350 bar) for three consecutive cycles to ensure complete cell wall rupture. The resulting suspension was centrifuged at 10,000× *g* for 20 min at 4 °C, and the supernatant was carefully collected. The clarified extract was then freeze-dried using a lyophilizer to obtain a dry NC aqueous extract powder, which was stored at −20 °C until further use.

### 2.3. AION Induction

Optic nerve head (ONH) ischemia was induced by intravenous injection of rose bengal (CAS No: 632-69-9, 2.5 M in phosphate-buffered saline; 1 mL/kg body weight; Sigma-Aldrich, St. Louis, MO, USA) via the tail vein. One minute after photosensitizer administration, laser photocoagulation was applied to the ONH. The ONH was exposed to an argon laser (532 nm wavelength) with a spot diameter of 80 μm. A total of 12 laser pulses were delivered, each with a duration of 1 s, using an OFA5.4 laser contact lens (Ocular Instruments Inc., Bellevue, WA, USA). The laser power was set at 80 mW (MC-500 Multicolor Laser, Nidek Co., Ltd., Tokyo, Japan). Following AION induction, animals were placed on a heating pad until full recovery from anesthesia. The NC extract solution was administered via subcutaneous injection immediately after AION induction and subsequently once daily for three consecutive days. In the sham group, animals received intravenous rose bengal administration but were not subjected to laser photoactivation of the optic nerve head; therefore, ischemic injury was not induced.

### 2.4. Flash Visual Evoked Potential (FVEP) Recording and Analysis

FVEP recordings were performed in accordance with our previously published protocols [23,24]. Anesthetized rats were secured in a stereotaxic frame (Stoelting, Wood Dale, IL, USA) for stable head positioning during recordings. Electrodes for recording were aligned over the frontal cortex (Bregma +1 mm) and the occipital cortex (8 mm posterior to bregma and 3 mm lateral), while the reference electrode was attached to the tail. FVEP responses were acquired using the Celeris system (Diagnosys LLC, Lowell, MA, USA). All recordings were conducted under dark-adapted conditions with background illumination turned off. Stimuli consisted of Ganzfeld flashes at an intensity of 0 dB delivered at a frequency of 1.9 Hz. For each recording, 100 sweeps were averaged. The artifact rejection threshold was set at 20 mV, and signals were sampled at 2000 Hz. The P1-N2 amplitude was measured and used for quantitative comparison of visual function among experimental groups.

### 2.5. Retrograde Labeling of Retinal Ganglion Cells with Fluoro-Gold (FG)

Retrograde labeling of retinal ganglion cells (RGCs) was performed as previously described [23,24]. Animals were anesthetized and fixed in a stereotaxic apparatus 21 days after induction of AION. Bilateral burr holes were created at the coordinates of 6 mm posterior to the bregma and 0.5 mm lateral to the midline. A total volume of 2 μL of 5% Fluoro-Gold (FG, RRID: AB_2314407) was injected into each superior colliculus using a Hamilton microsyringe. Seven days after FG injection, animals were euthanized and the eyeballs were enucleated. The globes were fixed in 4% paraformaldehyde (PFA, CAS No: 30525-89-4) in phosphate-buffered saline (PBS) (Acantor, Radnor, PA, USA) for 2 h at room temperature. Retinas were then carefully dissected and flat-mounted onto slides. Labeled RGCs were visualized using a fluorescence microscope (Axio Scope A1, Zeiss, Oberkochen, Germany) with appropriate filter settings (excitation: 350–400 nm; emission: 515 nm) and imaged using an Axiocam 705 color digital camera (Zeiss). Measurements of RGC density were performed at defined locations 1 mm and 3 mm from the ONH, corresponding to central and mid-peripheral regions of the retina. Eight randomly selected fields per retina were captured in both regions. RGC density was quantified from the acquired images using ImageJ software (version 1.54g, NIH, USA).

### 2.6. Preparation of Retinal and Optic Nerve Tissues

Following the FVEP recordings, rats were euthanized in a CO_2_ chamber with a controlled CO_2_ flow rate of 30% chamber volume per minute (approximately 5 L/min). The eyes, along with attached optic nerves, were immediately harvested. Tissues were immersed in 4% paraformaldehyde (PFA) and fixed at room temperature for 2 h. Following fixation, specimens were subjected to cryoprotection by sequential incubation in 10%, 20%, and 30% (*w*/*v*) sucrose solutions overnight. The eyes were then embedded in optimal cutting temperature (OCT) compound (SAKURA Finetek USA, Inc., Torrance, CA, USA) and stored at −80 °C prior to cryosectioning.

### 2.7. Terminal-Deoxynucleotidyl-Transferase-Mediated Nick End Labeling (TUNEL) Assay

Frozen retinal sections (8 μm thickness) were mounted onto slides and processed according to the manufacturer’s instructions for the Click-iT™ Plus TUNEL Assay for In Situ Apoptosis Detection (C10619, Invitrogen, Waltham, MA, USA). Sections were first rinsed with 1× phosphate-buffered saline (PBS) and fixed in 4% paraformaldehyde (PFA) for 15 min at room temperature. After washing with PBS, the tissues were permeabilized with proteinase K for 30 min at 37 °C. The sections were then incubated with the TUNEL reaction mixture to label DNA fragmentation associated with apoptosis. TUNEL-positive cells were visualized using a fluorescence microscope (Axio Scope A1, Zeiss, Oberkochen, Germany). Six randomly selected fields per section were imaged within the retinal layers. Quantification of apoptotic retinal ganglion cells (RGCs) was performed using ImageJ software.

### 2.8. Immunohistochemistry (IHC)

Following PBS rinsing, retinal and optic nerve sections were incubated in a blocking solution consisting of 1% BSA, 1% normal goat serum, and 1% Triton X-100. The sections were subsequently exposed to primary antibodies overnight at 4 °C, including IL-6 (1:200; ab9324, Abcam, Cambdrige, UK), Iba-1 (1:200; ab178846, Abcam), Ym1+2 (1:50; ab192029, Abcam), CNPase (1:200; ab6319, Abcam), and ED-1 (1:50; MCA341GA, Bio-Rad, Hercules, CA, USA). After primary antibody incubation, sections were washed twice with PBS and incubated with Alexa Fluor-conjugated secondary antibodies (Invitrogen, Waltham, MA, USA) for 1 h at room temperature in the dark. Nuclear counterstaining was performed with DAPI (1:500, Sigma, St. Louis, MO, USA). Fluorescent images were acquired using a fluorescence microscope (Axio Scope A1, Zeiss, Oberkochen, Germany). Images were acquired from six randomly selected fields per section in both retinal and optic nerve areas. Quantification of CNPase expression was performed by measuring the immunopositive area in ImageJ and normalizing it to the corresponding number of DAPI-stained nuclei.

### 2.9. Immunoblot Analysis

Retinal tissues (*n* = 6) were homogenized in RIPA buffer supplemented with protease and phosphatase inhibitors (#78442, Invitrogen, Waltham, MA, USA) using a sonicator (Sonics & Materials, Inc., Newtown, CT, USA) for protein extraction. Lysates were centrifuged at 13,500× *g* for 15 min at 4 °C, and the supernatants were collected for subsequent Western blot analysis. Equal amounts of protein (30 μg per lane) were separated by SDS-PAGE and transferred onto 0.45 μm polyvinylidene difluoride (PVDF) membranes. Membranes were blocked with 5% non-fat milk in TBST for 1 h at room temperature and then incubated overnight at 4 °C with the following primary antibodies: PI3K (1:500, RRID: AB_632211, sc-423, Santa Cruz, CA, USA), phospho-PI3K (1:200, RRID: AB_2252313, sc-12929, Santa Cruz, USA), Akt (1:500, RRID: AB_1147620, #2920, Cell Signaling, Danvers, MA, USA), phospho-Akt (1:500, RRID: AB_2315049, #4060, Cell Signaling, USA), mTOR (1:200, RRID: AB_2882219, #66888, Proteintech, Rosemont, IL, USA), phospho-mTOR (1:200, RRID: AB_3673645, #67778, Proteintech, USA), Rictor (1:200, RRID: AB_2179963, #2114, Cell Signaling, USA), p70S6 kinase (1:200, RRID: AB_2943679, #34475, Cell Signaling, USA), and phospho-p70S6 kinase (1:200, RRID: AB_2295301, #2934, Cell Signaling, USA). After primary antibody incubation, membranes were washed three times with TBST and incubated with horseradish peroxidase-conjugated secondary antibodies (1:10,000 dilution in 5% non-fat milk) for 1 h at room temperature. Glyceraldehyde-3-phosphate dehydrogenase (GAPDH, 1:10,000, RRID: AB_1078991, G8795, SIGMA, Scottsdale, AZ, USA) was used as the internal loading control. Protein signals were detected using an enhanced chemiluminescence (ECL) system (RPN2232, Cytiva, Marlborough, MA, USA), and images were captured using the iBright CL750 Imaging System (Thermo Fisher Scientific, Waltham, MA, USA). For each experiment, three retinal samples per group were pooled, and all experiments were independently repeated three times to ensure reproducibility.

### 2.10. Proteomic Characterization of Nostoc commune Aqueous Extract

#### 2.10.1. Protein Extraction and Sample Preparation

Lyophilized *Nostoc commune* (NC) aqueous extract was reconstituted in lysis buffer containing 8 M urea, 50 mM Tris-HCl (pH 8.0), and a protease inhibitor cocktail. The suspension was sonicated on ice and centrifuged at 14,000× *g* for 20 min at 4 °C to remove insoluble debris. Protein levels were measured using a bicinchoninic acid (BCA) assay.

#### 2.10.2. Trypsin Digestion and LC–MS/MS Analysis

Tryptic peptides were diluted in HPLC buffer A (0.1% formic acid in water) and loaded onto a reverse-phase trap column (Zorbax 300SB-C18, 0.3 × 5 mm; Agilent Technologies, Santa Clara, CA, USA) for desalting. Peptides were subsequently separated on a home-packed analytical column (Waters BEH C18, 1.7 μm particle size, 100 μm inner diameter × 10 cm length with a 15 μm emitter tip) using a 70 min gradient of HPLC buffer B (99.9% acetonitrile containing 0.1% formic acid) at a flow rate of 0.3 μL/min. The LC system was coupled to a 2D linear ion trap–Orbitrap mass spectrometer (Orbitrap Elite ETD; Thermo Fisher Scientific, Waltham, MA, USA) operated with Xcalibur software (version 2.2). Full MS scans were acquired in the Orbitrap over an m/z range of 400–2000 at a resolution of 120,000 (m/z 400). Internal calibration was performed using the lock-mass ion m/z 536.165365. Data-dependent acquisition consisted of one MS survey scan followed by twenty MS/MS scans of the most abundant precursor ions. Dynamic exclusion was set to 40 s with a mass tolerance window of 15 ppm. The electrospray voltage was 2.0 kV and the capillary temperature was maintained at 200 °C. Automatic gain control targets were set to 3 × 10^6^ ions for MS scans and 3 × 10^3^ ions for MS/MS scans, with maximum injection times of 1000 ms and 200 ms, respectively.

#### 2.10.3. Protein Identification and Quantification

Raw MS/MS data were processed using Proteome Discoverer (version 2.3; Thermo Fisher Scientific). Spectra were searched against the UniProt Nostoc protein database using the Mascot search engine (version 2.5; Matrix Science, London, UK). The precursor mass tolerance was set to 10 ppm and the fragment ion tolerance to 0.5 Da. Trypsin was specified as a digestion enzyme with up to two missed cleavages. Methionine oxidation and protein N-terminal acetylation were defined as variable modifications, whereas carbamidomethylation of cysteine was set as a fixed modification. Peptide-spectrum matches were filtered using Mascot rank-1 identifications with an overall false discovery rate (FDR) < 1%. Proteins identified by a single peptide were excluded, and only proteins supported by at least two unique peptides were retained. Relative protein abundance was estimated using normalized spectral abundance values derived from peptide-spectrum match counts. The mass spectrometry proteomics data have been deposited to the ProteomeXchange Consortium via the PRIDE [25] partner repository with the dataset identifier PXD076497 and 10.6019/PXD076497.

### 2.11. Statistical Analysis

Statistical analyses were performed using Prism 9.0 software (GraphPad Software, San Diego, CA, USA). Comparisons among more than two experimental groups were conducted using a non-parametric Kruskal–Wallis test. When a significant overall effect was observed, post hoc multiple comparisons between groups were performed using Dunn’s test with adjustment for multiple comparisons. All quantitative data are presented as mean ± standard deviation (SD). A two-tailed *p*-value of <0.05 was considered statistically significant.

## 3. Results

### 3.1. NC Extract Promotes RGC Survival Following Ischemic Optic Nerve Injury

**Figure 1:**
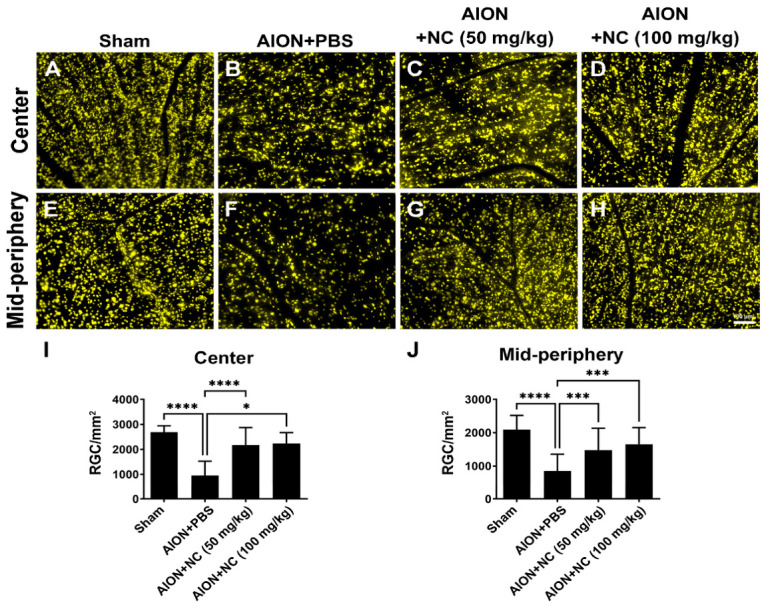
Morphology and quantification of retinal ganglion cells (RGCs) in the central and mid-peripheral retina following ischemic optic injury. (**A**–**D**) Representative FG-labeled images of the central retina show the protective effect of NC extract. The calculated RGC survival rates in this region were 35.4% in the AION + PBS group, significantly increasing to 80.9% and 83.2% in the AION + 50 mg/kg and AION + 100 mg/kg NC extract groups, respectively. (**E**–**H**) Representative images of the mid-peripheral retina following Fluoro-Gold (FG) labeling are presented. RGC survival rates in this mid-peripheral region were 40.6% in the AION + PBS group, increasing significantly to 70.5% and 70.9% in the AION + 50 mg/kg and AION + 100 mg/kg NC extract groups, respectively. (**I**,**J**) Quantification of RGC density (RGCs/mm^2^) across the central and mid-peripheral areas of the whole-mounted retinas. (A–H, same magnification; scale bar = 100 μm, *n* = 6 in each group, and * *p* < 0.05, *** *p* < 0.001, and **** *p* < 0.0001).

Fluoro-Gold (FG) has been widely used as a retrograde tracer to label functional neurons in the central nervous system [26]. In this study, functional retinal ganglion cells (RGCs) were identified following ischemic injury using FG retrograde labeling to visualize neuronal soma. Quantitative analysis demonstrated that, in the central retina, RGC densities in the sham, AION + PBS, AION + 50 mg/kg NC extract, and AION + 100 mg/kg NC extract groups were 2691 ± 255.6, 952.1 ± 576.9, 2177 ± 693.9, and 2239 ± 432.9 RGCs/mm^2^, respectively (Figure 1A–D,I). In the mid-peripheral retina, RGC densities were 2095 ± 432, 850.9 ± 504.3, 1478 ± 655.3, and 1654 ± 498.4 RGCs/mm^2^ in the respective groups (Figure 1E–H,J). These FG retrograde labeling results indicate that NC extract treatment significantly preserves RGC survival following ischemic insult.

### 3.2. NC Extract Administration Recused the Visual Function

**Figure 2:**
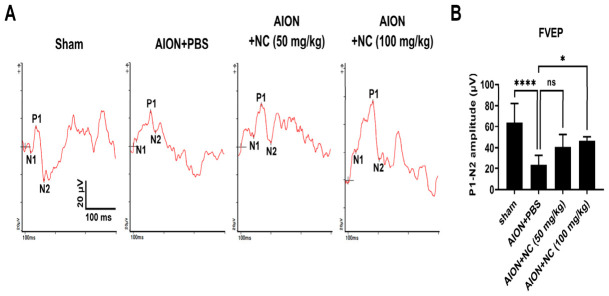
A 100 mg/kg dose of NC extract can effectively restore visual function after ischemic optic neuropathy. (**A**) Representative traces of Flash Visual Evoked Potentials (FVEPs) obtained 28 days post-AION induction are shown. (**B**) The bar graph illustrates the quantification of the P1-N2 amplitudes. The recovered visual function rates, based on the P1-N2 amplitudes, were 37.1% for the PBS-treated group, 63.8% for the AION + 50 mg/kg NC extract group, and 72.8% for the AION + 100 mg/kg NC extract group, respectively. (*X*-axis represents time, 0–100 ms; *Y*-axis represents amplitude, 20 μV; *n* = 6 in each group, ns, not significant; * *p* < 0.05, and **** *p* < 0.0001).

FVEPs were recorded at 28 days following ischemic induction. The P1–N2 amplitudes in the sham, AION + PBS, AION + 50 mg/kg NC extract, and AION + 100 mg/kg NC extract groups were 63.9 ± 18.1 μV, 23.7 ± 8.8 μV, 40.8 ± 11.8 μV, and 46.5 ± 4.1 μV, respectively (Figure 2). Notably, treatment with 100 mg/kg NC extract significantly improved visual function compared with PBS-treated controls. This effect was further supported by RGC morphometric and functional assessments, indicating a significant therapeutic benefit at this dose. Based on these findings, a dose of 100 mg/kg NC extract was selected for subsequent experiments.

### 3.3. NC Extract Treatment Reduced RGC Apoptosis in the Retina

**Figure 3:**
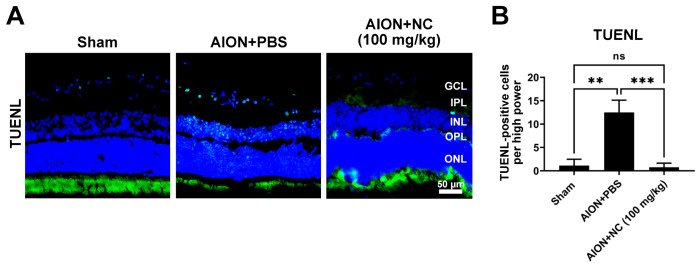
NC extract reduces apoptotic cell death in the retinal ganglion cell layer (GCL). (**A**) Representative images of retinal sections stained with TUNEL in the three experimental groups: Sham, AION + PBS, and AION + 100 mg/kg NC. DAPI (blue) labels cell nuclei, and TUNEL staining (green) indicates apoptotic-positive cells in the retina. (**B**) Quantitative analysis of TUNEL-positive cells in the retinal ganglion cell layer. The number of apoptotic-positive cells per high-power field (HPF) was 1.1 ± 1.4 in the sham group, significantly increased to 12.5 ± 2.6 in the AION + PBS group, and markedly reduced to 0.8 ± 0.9 in the AION + 100 mg/kg NC group (Scale bar = 50 μm, *n* = 6 per group; ns, not significant; ** *p* < 0.01; *** *p* < 0.001).

Apoptotic and necrotic processes are well-recognized mechanisms underlying RGCs death following partial optic nerve injury [27,28]. To evaluate apoptosis in the retinal ganglion cell layer (RGCL) after ischemic induction, a terminal deoxynucleotidyl transferase dUTP nick end labeling (TUNEL) assay was performed to detect DNA fragmentation in retinal sections. The number of TUNEL-positive cells in the ganglion cell layer (GCL) was significantly decreased in the NC extract-treated group compared with the PBS-treated group following AION induction (Figure 3). These results indicate that NC extract administration attenuates apoptosis in the retina after ischemic injury, thereby contributing to enhanced RGC survival.

### 3.4. NC Extract Restrained Macrophage Infiltration in the Optic Nerve

**Figure 4:**
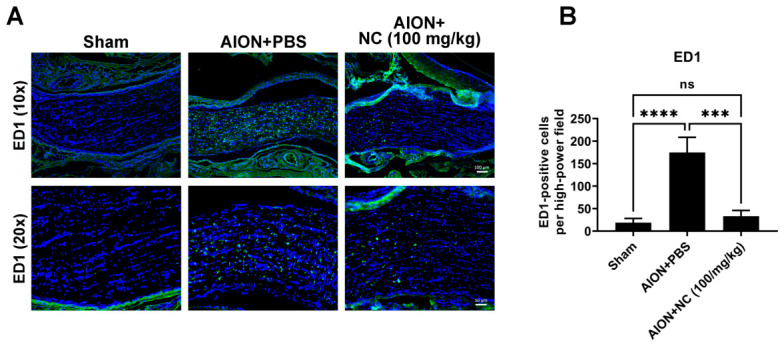
NC extract treatment significantly reduces macrophage infiltration following ischemic optic neuropathy. (**A**) Representative immunostaining displays representative images of the optic nerve fields stained for ED-1 (green), a marker for activated phagocytic cells, and DAPI (blue), a marker for cell nuclei, across the three groups: Sham, AION + PBS, and AON + 100 mg/kg NC group. (**B**) The quantitative analysis of ED-1-positive cells in the optic nerve. The number of ED-1-positive cells per high-power field (HPF) was found to be 18.8 ± 9.3 in the sham group. This count dramatically increased in the AION + PBS group (174.7 ± 33.8), but was significantly reduced in the AION + 100 mg/kg NC extract group (33.2 ± 13.0) (Scale bars: 100 μm (upper), 50 μm (lower). *n* = 6 per group. ns, not significant; *** *p* < 0.001, **** *p* < 0.0001).

In previous studies, inflammatory cell infiltration following ischemic optic neuropathy has been identified [9,29]. ED-1 is a recognized marker for activated phagocytic cells involved in inflammation [30]. To assess macrophage infiltration into the optic nerve, ED-1 was used to label activated macrophages after ischemic optic injury. Compared with the PBS-treated group, NC extract treatment resulted in a significant reduction in activated macrophage infiltration in the optic nerve (Figure 4). These data suggest that NC extract may modulate inflammation following ischemic insult.

### 3.5. NC Extract Suppresses Pro-Inflammatory Cytokine Expression While Promoting Anti-Inflammatory Responses

**Figure 5:**
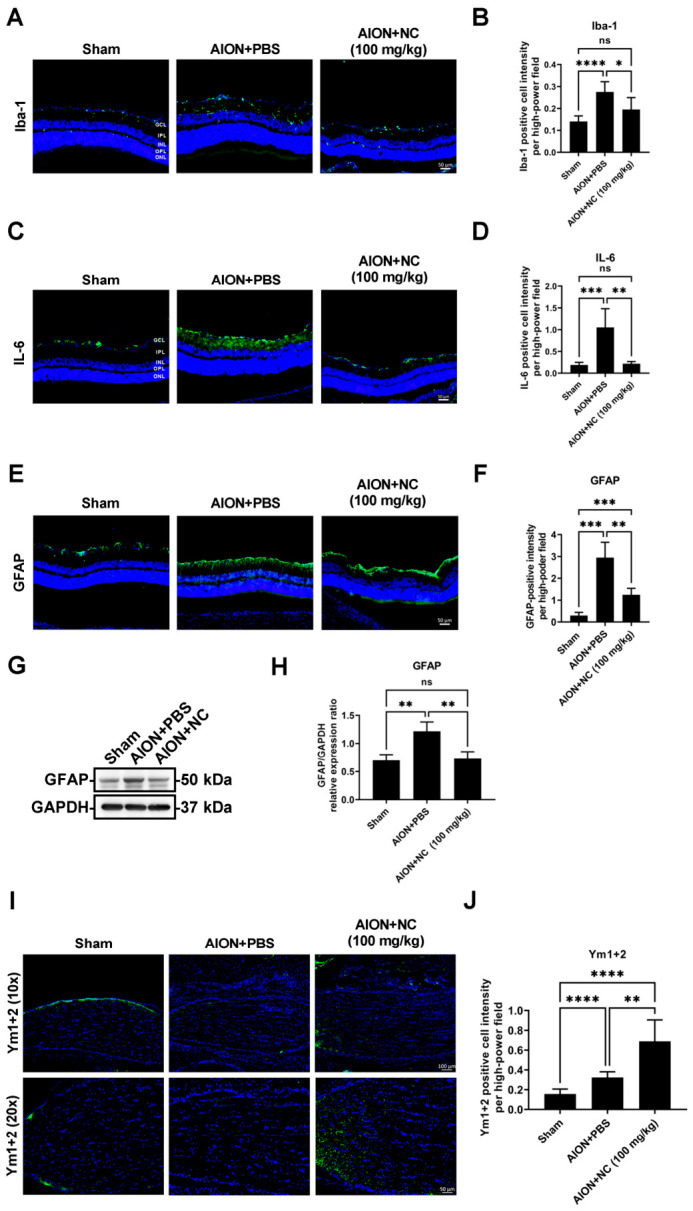
NC extract modulates neuroinflammatory markers in the retina and optic nerve following AION induction. Fluorescent labeling of Iba1, IL-6, GFAP, and Ym1+2 in retinal and optic nerve sections is shown following AION induction. Retinal GFAP expression was further assessed by immunoblotting at 28 days post-injury. (**A**) Fluorescent labeling of Iba1 (green) and DAPI (blue), a nuclear marker, in retinal sections from sham, AION + PBS, and AION + NC-treated animals. (**B**) Quantitative analysis of Iba1-positive intensity showed values of 0.14 ± 0.03, 0.28 ± 0.05, and 0.20 ± 0.05 per high-power field (HPF) in the sham, AION + PBS, and AION + NC groups, respectively (scale bar = 50 μm; *n* = 6 per group; ns, not significant; * *p* < 0.05; and **** *p* < 0.0001). (**C**) IL-6 immunofluorescence (green) and DAPI (blue), a nuclear marker, in retinal sections from the sham, AION + PBS, and AION + NC groups. (**D**) Quantification of IL-6-positive intensity revealed values of 0.19 ± 0.06, 1.05 ± 0.43, and 0.22 ± 0.05 per HPF in the sham, AION + PBS, and AION + NC groups, respectively (scale bar = 50 μm; *n* = 6 per group; ns, not significant; ** *p* < 0.01, and *** *p* < 0.001). (**E**) GFAP immunofluorescence (green) and DAPI (blue), a nuclear marker, in retinal sections from the sham, AION + PBS, and AION + NC groups at 28 days post-rAION. (**F**) Quantitative analysis of GFAP-positive intensity demonstrated values of 0.30 ± 0.14, 2.95 ± 0.70, and 1.25 ± 0.30 per HPF in the sham, AION + PBS, and AION + NC groups, respectively (scale bar = 50 μm; *n* = 6 per group; ** *p* < 0.01; and *** *p* < 0.001). (**G**) Representative immunoblot showing GFAP protein expression in retinal tissue 28 days after rAION induction. (**H**) Densitometric analysis of GFAP protein levels showed relative expression values of 0.70 ± 0.10, 1.21 ± 0.16, and 0.72 ± 0.12 in the sham, AION + PBS, and AION + NC groups, respectively. (ns, not significant; ** *p* < 0.01). (**I**) Ym1/2 immunofluorescence (green) and DAPI (blue), a nuclear marker, in optic nerve sections from the sham, AION + PBS, and AION + NC groups. (**J**) Quantitative analysis of Ym1/2-positive cells in the optic nerve demonstrated values of 0.16 ± 0.05, 0.32 ± 0.06, and 0.69 ± 0.22 in the sham, AION + PBS, and AION + NC groups, respectively (Scale bars: 100 μm (upper), 50 μm (lower). *n* = 6 per group. ** *p* < 0.01, and **** *p* < 0.0001).

The post-stroke immune response has emerged as a promising therapeutic target for ischemic stroke. In the central nervous system (CNS), glial cells—including microglia, astrocytes, and oligodendrocytes—constitute key components of the peri-infarct microenvironment and play critical roles in modulating post-ischemic immune responses [31,32]. To characterize neuroinflammatory responses in ischemic optic neuropathy, markers associated with M1 and M2 glial phenotypes, including GFAP, Iba-1, IL-6, and Ym1+2, were examined in retinal and optic nerve sections using immunohistochemical (IHC) analysis [33]. IHC results demonstrated a reduction in Iba1-positive microglial cells in the retina following NC extract treatment (Figure 5A,B). Retinal IL-6 levels, a pro-inflammatory cytokine, were significantly reduced in the NC-treated group **(**Figure 5C,D). GFAP protein expression, indicative of reactive gliosis, was also markedly reduced in the retina after NC extract administration (Figure 5E–H). In contrast, expression of the anti-inflammatory marker Ym1+2 was significantly increased in the optic nerve following NC extract treatment (Figure 5I,J). Collectively, these findings suggest that NC extract modulates neuroinflammatory responses by suppressing pro-inflammatory signaling and promoting anti-inflammatory polarization following AION induction.

### 3.6. NC Extract Attenuates Demyelination Following Ischemic Injury

**Figure 6:**
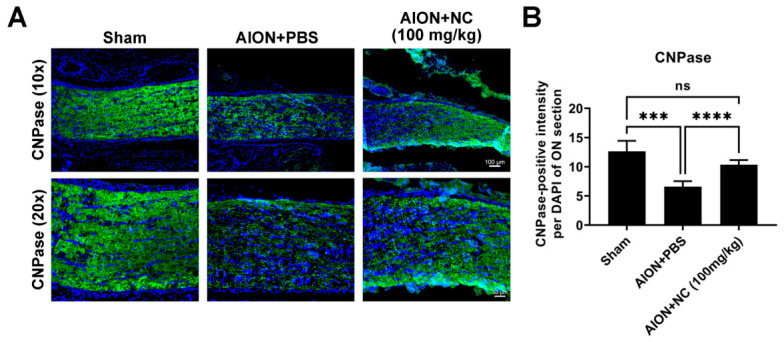
NC Extract preserves myelination in the optic nerve following AION induction. (**A**) The protective effect of NC extract on myelin structure, measured 28 days after rAION induction, is illustrated by the representative images of optic nerve sections stained for CNPase (green) and DAPI (blue), a nuclear marker, in the Sham, AION + PBS, and AION + NC extract groups. (**B**) Quantitative analysis of the CNPase-positive intensity normalized to DAPI revealed significant differences among the groups. The intensity was measured as 12.64 ± 1.79 in the Sham group, dropped markedly to 6.57 ± 0.96 in the AION + PBS group, and was partially restored to 10.35 ± 0.77 CNPase-positive intensity/DAPI in the AION + NC group (Scale bars: 100 μm (upper), 50 μm (lower). *n* = 6 per group. ns, not significant; *** *p* < 0.001, **** *p* < 0.0001).

In the central nervous system (CNS), oligodendrocytes are responsible for the formation and maintenance of the myelin sheath, which insulates axons and facilitates saltatory conduction to enable rapid transmission of action potentials [34]. Oligodendrocytes are particularly susceptible to ischemic injury, and myelin loss represents a hallmark pathological feature of white matter stroke (WMS) [35]. To assess demyelination in the optic nerve following ischemic injury, CNPase (2′,3′-cyclic nucleotide 3′-phosphodiesterase), a myelin-associated enzyme and established marker of oligodendrocytes, was used to evaluate myelin integrity. Previous studies have demonstrated that alterations in CNPase expression are associated with myelin structural disruption [36]. Immunohistochemical analysis revealed that NC extract treatment significantly preserved CNPase expression and restored myelin structure compared with the PBS-treated group (Figure 6A,B). These findings suggest that NC extract confers protective effects against ischemia-induced demyelination, thereby contributing to the maintenance of axonal integrity following ischemic insult.

### 3.7. NC Extract Inhibits Apoptosis and Activates the PI3K/AKT/mTOR/p70S6K Signaling Pathway Following Ischemic Optic Neuropathy

**Figure 7:**
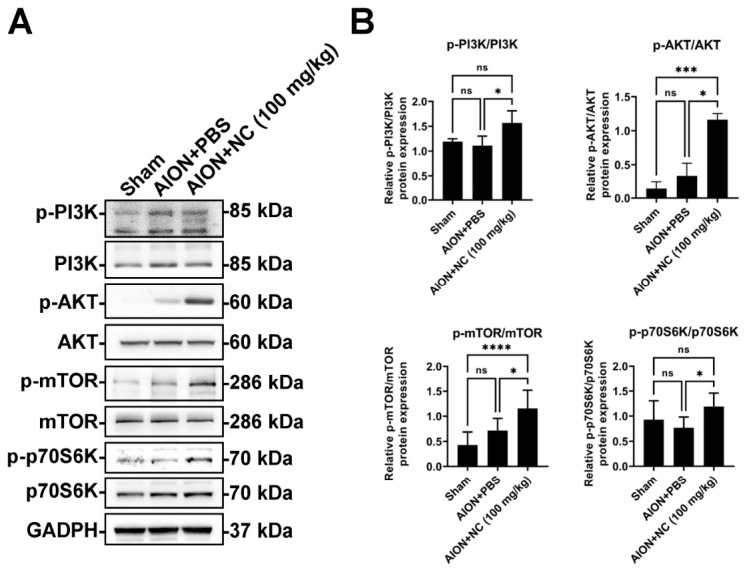
NC extract attenuates apoptosis and is associated with activation of the PI3K/AKT/mTOR/p70S6K signaling pathway following ischemic optic injury. (**A**) Representative Western blot images showing increased phosphorylation levels of PI3K, AKT, mTOR, and p70S6K in the NC extract-treated group compared with the PBS-treated group after ischemic optic injury. (**B**) Quantitative analysis of Western blot band intensity is shown. Data are presented as mean ± SD and were normalized to the GAPDH loading control. (*n* = 6 per group; * *p* < 0.05; *** *p* < 0.001; **** *p* < 0.0001).

To elucidate the molecular mechanisms underlying the protective effects of NC extract on retinal ganglion cell (RGC) survival and visual function after ischemic insult, the PI3K/AKT/mTOR signaling cascade was examined by Western blot analysis. The results demonstrated that the levels of phosphorylated PI3K, AKT, and mTOR were significantly elevated in the NC extract-treated group compared with the PBS-treated group. In addition, enhanced mTOR phosphorylation was accompanied by increased activation of its downstream effector, 70 kDa ribosomal protein S6 kinase (p70S6K), in the NC-treated group (Figure 7A,B). These findings suggest that NC extract treatment is accompanied by activation of the PI3K/AKT/mTOR/p70S6K signaling pathway and reduced apoptosis following ischemic optic neuropathy.

### 3.8. Proteomic Profile of Nostoc commune Aqueous Extract

Proteomic profiling of the Nostoc commune (NC) aqueous extract revealed a highly structured and functionally coherent protein landscape dominated by photosynthetic antenna complexes, antioxidant enzymes, metabolic regulators, and stress-response factors. Among the proteins detected, those exceeding 1% relative abundance displayed consistently high sequence coverage, strong Mascot scores, and robust peptide-spectrum matches (PSMs), supporting the reliability of their identification (Table 1).

The most abundant proteins were phycobiliproteins, including phycocyanin α- and β-chains (CpcA, CpcB), allophycocyanin subunits (ApcA, ApcB), phycoerythrin chains (CpeA, CpeB), and associated linker proteins (CpcC, CpcG). Collectively, these light-harvesting proteins accounted for a substantial proportion of the total proteomic signal, with individual relative abundances ranging from approximately 2–9% per protein. This enrichment is consistent with the known antioxidant and radical-scavenging properties of cyanobacterial phycobiliproteins [37,38,39,40,41,42,43].

Beyond photosynthetic components, robust antioxidant and cytoprotective enzymes were prominently represented. These included superoxide dismutase (SOD) [20,44], Mn-containing catalase [45,46,47], and peroxiredoxin (PRDX2-4) [48,49,50,51], together comprising a significant fraction of the extract proteome. The concurrent detection of several ROS-detoxification–related enzymes in the proteomic analysis suggests that the NC extract contains proteins potentially involved in oxidative stress regulation.

Proteins involved in cellular stress tolerance and genome protection, such as DNA-binding starvation proteins (Dps) [52], RNA-binding proteins and DNA repair-associated enzymes were also consistently detected. In addition, key metabolic enzymes—including glutamine synthetase, ferredoxin–NADP reductase, 6-phosphogluconate dehydrogenase, ATP synthase subunit β, and inorganic pyrophosphatase—suggest preserved metabolic functionality and redox balance within the extract. Notably, several signal-transduction-related proteins, including histidine kinases and oxidoreductases, were identified. Together, these findings suggest that NC extract contains stress-response, metabolic, and signaling proteins detected by proteomic analysis, which may contribute to the observed biological effects of the extract.

## 4. Discussion

**Figure 8:**
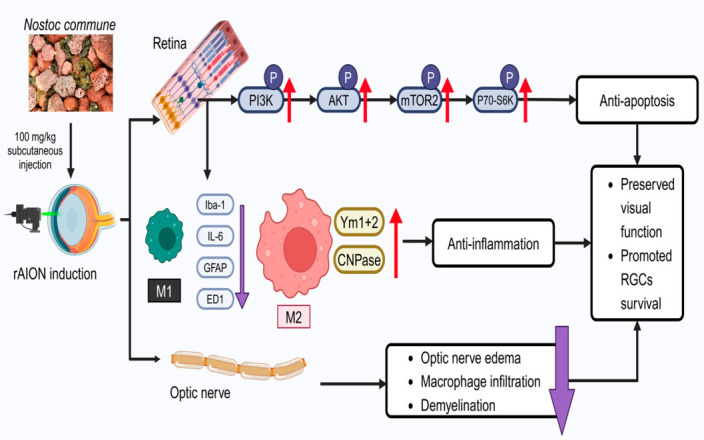
Proposed neuroprotective mechanism of Nostoc commune extract in rAION. Schematic illustration showing that NC extract (100 mg/kg) activates PI3K/AKT/mTOR/p70S6K signaling, suppresses apoptosis and inflammation, promotes M2-associated and myelin-related markers (Ym1+2 and CNPase), and reduces optic nerve edema, macrophage infiltration, and demyelination after rAION induction. These effects are associated with preserved visual function and enhanced retinal ganglion cell survival.

In this study, we demonstrated the neuroprotective effects of NC extract in an rAION model, highlighting its role in reducing inflammatory cytokine release, promoting myelination, and involving specific molecular signaling pathways. NC extract enhanced RGC survival and preserved visual function accompanied by activation of the PI3K/AKT/mTOR/p70S6K signaling pathway. These findings confirm the beneficial effects of NC extract on ocular health (Figure 8).

The immunoblot findings indicate that NC treatment is accompanied by increased activation of the PI3K/AKT/mTOR signaling pathway, together with enhanced RGC survival and preservation of visual function following ischemic optic neuropathy. The mammalian target of rapamycin (mTOR) is a 289 kDa serine/threonine kinase belonging to the phosphoinositide 3-kinase (PI3K)-related kinase family and is highly conserved across species [53]. Dysregulation of the mTOR signaling pathway has been implicated in a wide range of pathological conditions, including cancer, neurodegenerative disorders, and diabetes mellitus [54,55]. As a specialized component of the central nervous system, the retina comprises complex neural circuits responsible for processing visual information and transmitting electrical signals to the brain [56]. Previous studies have demonstrated that the PI3K/Akt/mTOR pathway is crucial for the survival of retinal progenitor cells under hypoxic and oxidative stress conditions, thereby protecting these cells from apoptosis. Furthermore, docosahexaenoic acid (DHA) regulates oxidative stress-induced apoptosis in human retinal pigment epithelial (RPE) cells through the PI3K/Akt/mTOR/p70S6K signaling pathways. Moreover, docosahexaenoic acid (DHA) has been shown to attenuate oxidative stress-induced apoptosis in human retinal pigment epithelial (RPE) cells via the PI3K/AKT/mTOR/p70S6K signaling cascade [57]. These findings suggest that activation of the PI3K/AKT/mTOR/p70S6K signaling pathway is associated with reduced apoptosis, improved RGC survival, and the neuroprotective effects of Nostoc commune in ischemic optic neuropathy.

Previous studies have documented the presence of ED1-positive cells following ischemic injury to the optic nerve, reflecting activation of microglia and infiltrating macrophages and subsequent release of proinflammatory cytokines that initiate and propagate inflammatory responses [58,59]. During the acute phase of ischemic stroke, reactive oxygen species (ROS) and proinflammatory mediators, including ED1, Iba-1, IL-6, IL-1β, GFAP, and various chemokines, are rapidly upregulated within injured tissue. These events contribute to disruption of the blood–optic nerve barrier, optic nerve edema, and progressive neuronal degeneration [60]. In the present study, immunoblot and immunohistochemical analyses demonstrated that NC extract administration significantly reduced IL-6 expression and the accumulation of Iba1-positive cells, as well as attenuated GFAP expression in the retina. These findings indicate suppression of proinflammatory signaling and reactive gliosis following ischemic injury. In addition, NC extract enhanced Ym1+2 expression in the optic nerve. Ym1+2 is a marker associated with alternatively activated (M2) microglia during the anti-inflammatory phase after ischemic insult [61]. Our immunoblot and immunohistochemical analyses demonstrated that NC extract administration significantly reduced IL-6 expression and the accumulation of Iba1-positive cells, as well as attenuated GFAP expression in the retina. These findings indicate suppression of proinflammatory signaling and reactive gliosis following ischemic injury. In addition, NC extract enhanced Ym1+2 expression in the optic nerve. Ym1+2 is a marker associated with alternatively activated (M2) microglia during the anti-inflammatory phase after ischemic insult [62]. The evidence suggests that promoting M2 microglial polarization is a critical therapeutic strategy in neurodegenerative diseases. Facilitating the phenotypic shift in microglia from the proinflammatory M1 state to the anti-inflammatory M2 state has been proposed as a promising approach for conditions such as Alzheimer’s disease (AD), Parkinson’s disease (PD), amyotrophic lateral sclerosis (ALS), and multiple sclerosis (MS) [63,64,65]. These observations raise the possibility that NC extract may influence microglial polarization toward an M2 phenotype, thereby contributing to an anti-inflammatory and immunoregulatory microenvironment.

Inflammation contributes to axonal demyelination in the optic nerve, which subsequently leads to RGC apoptosis [66]. Clinical and experimental studies have further demonstrated that thinning of the retinal nerve fiber layer (RNFL), a surrogate marker of RGC axonal loss, is strongly associated with visual impairment following optic nerve injury [24,67,68]. Our immunohistochemical analysis revealed that NC extract treatment preserved axonal myelination in the optic nerve after ischemic injury, indicating protection against inflammation-associated demyelination. These findings confirm the neuroprotective effects of NC extract in the rAION model, including attenuation of RGC apoptosis and suppression of neuroinflammatory responses. NC extract promoted RGC survival and preserved visual function following rAION induction through activation of the PI3K/AKT/mTOR/p70S6K signaling pathway and modulation of microglial polarization toward an anti-inflammatory M2 phenotype. This effect was accompanied by reduced expression of proinflammatory and glial activation markers (Iba-1, IL-6, and GFAP) and increased expression of the M2-associated marker Ym1+2 in the retina and optic nerve. Increasing evidence indicates that the PI3K/AKT/mTOR axis is a key regulatory pathway in ischemic and neurodegenerative disorders, where it suppresses apoptosis and modulates oxidative stress and inflammatory responses. In ischemic stroke, activation of PI3K/Akt signaling has been reported to protect neurons against hypoxic/ischemic damage and to interact with mTOR-dependent pathways involved in cell survival and autophagy regulation [69]. In neurodegenerative diseases such as Parkinson’s disease, PI3K/AKT/mTOR signaling is closely associated with the control of oxidative stress through downstream mediators, including GSK-3β and FoxO3a [70].

Recent studies have also reported that Nostoc commune extract exhibits significant antioxidant and anti-inflammatory activities in neurological disease models. For example, NC extract was shown to alleviate oxidative stress and neuroinflammatory responses in a mouse model of schizophrenia, suggesting its potential neuroprotective properties in central nervous system disorders [20,40]. These findings support the present observations that NC extract suppresses neuroinflammation and oxidative stress following ischemic optic injury. Consistent with these observations, our results demonstrate that NC extract activates the PI3K/AKT/mTOR/p70S6K signaling pathway to inhibit apoptosis and suppress inflammatory responses, thereby promoting RGC survival following ischemic optic neuropathy.

From a food and function perspective, the proteomic composition of *Nostoc commune* aqueous extract indicates that its biological activity is driven by a concerted network of food-derived proteins rather than a single dominant bioactive compound. The high abundance of phycobiliproteins, together with endogenous antioxidant enzymes, supports the role of NC as a functional food ingredient with intrinsic oxidative stress-modulating capacity [39,41,71]. Oxidative stress and low-grade inflammation are widely recognized contributors to age-related visual decline and ischemia-associated neural dysfunction. The presence of multiple redox-regulating proteins in NC extract suggests that its consumption or administration may help support cellular antioxidant defenses and inflammatory balance, consistent with the observed reductions in inflammatory markers and apoptotic indices in vivo.

Proteomic profiling of the NC extract revealed several functional protein groups that may contribute to its biological activity. These include phycobiliproteins such as phycocyanin (CpcA and CpcB), allophycocyanin (ApcA and ApcB), and phycoerythrin (CpeA and CpeB), which have been reported to exhibit antioxidant and anti-inflammatory activities [41,72]. In addition, antioxidant enzymes, including superoxide dismutase, Mn-containing catalase, and peroxiredoxins (PRDX2-4), were detected, suggesting the presence of components involved in reactive oxygen species (ROS) detoxification that protects cells from oxidative damage [47]. Several stress-adaptive and metabolic proteins were also identified, including the starvation-inducible DNA-binding protein (Dps), glutamine synthetase, ferredoxin-NADP reductase, and 6-phosphogluconate dehydrogenase, which are involved in cellular stress adaptation, redox regulation, and metabolic homeostasis [52]. Additional metabolic enzymes, such as ATP synthase subunit beta, further suggest a role in maintaining cellular energy balance. Importantly, the presence of these antioxidant, stress-adaptive, and metabolic proteins suggests that NC extract may help maintain cellular homeostasis under metabolic challenge, a key concept in food-function science. Rather than acting as a direct pharmacological modulator, NC extract may improve the cellular microenvironment by enhancing redox balance and metabolic stability. Within this framework, activation of the PI3K/AKT/mTOR/p70S6K signaling pathway observed in retinal tissue may represent a secondary adaptive response to improved cellular conditions. This coordinated regulation of oxidative stress, inflammation, and metabolism may contribute to the observed neuroprotective effects for the observed neuroprotective effects of NC extract in the rAION model.

Collectively, these findings position *Nostoc commune* as a protein-rich functional food candidate with meaningful potential to support ocular health, particularly under conditions of ischemic or oxidative stress. The diverse repertoire of antioxidant and cytoprotective proteins identified in the extract suggests that its biological effects arise from a coordinated network rather than a single dominant component. This multimodal profile aligns well with the growing recognition that complex natural products can enhance tissue resilience through simultaneous modulation of redox balance, inflammation, and cellular survival pathways. Looking ahead, systematic evaluation of the digestion stability, bioaccessibility, and structure–function relationships of key *Nostoc* proteins will be essential for establishing its utility as a standardized functional ingredient. Such efforts will not only clarify its translational relevance but may also help lay the groundwork for developing nutritionally derived strategies to preserve visual function in individuals at risk of ischemic or oxidative retinal injury.

Despite the promising findings of this study, several limitations should be acknowledged. First, the present study was conducted using a rodent rAION model, and the extent to which these results translate to primate or human NAION remains uncertain. Second, the NC preparation used in this study was a crude aqueous extract; therefore, the specific bioactive components responsible for the observed neuroprotective effects have not yet been fully identified. Future studies should focus on isolating and characterizing the active constituents and determining their individual contributions to the observed therapeutic effects. Third, although NC extract demonstrated beneficial effects in this model, further investigations are required to optimize the dosage regimen, route of administration, and therapeutic time window. In addition, pharmacokinetic properties and long-term safety profiles remain to be determined. Addressing these limitations will be essential for advancing the translational potential of NC extract as a therapeutic strategy for ischemic optic neuropathy.

## 5. Conclusions

Taken together, our findings demonstrate that aqueous *Nostoc commune* extract exerts robust neuroprotective effects in the rAION model, preserving retinal ganglion cell integrity and visual function. These effects are associated with attenuation of apoptotic and neuroinflammatory responses, reprogramming of glial activity toward a reparative phenotype, and preservation of optic nerve structure, accompanied by activation of the PI3K/AKT/mTOR signaling pathway. Together, these multimodal structural, molecular, and functional improvements highlight NC as a promising phytotherapeutic candidate for ischemic optic neuropathy. Future investigations should focus on identifying active components, elucidating pharmacokinetic and safety profiles, optimizing delivery strategies, and validating therapeutic efficacy in translational settings.

## Figures and Tables

**Table 1:** Major proteins identified in Nostoc commune aqueous extract and their functional classification.

Accession	Protein	Coverage [%]	Mascot Score	PSMs	Relative Abundance (%)	Functional Category
A0A5P8W0R1	CpcA, phycocyanin alpha chain	80	8680	194	9.39%	Phycobiliprotein/Antioxidant
A0A5P8W0X3	CpcB, phycocyanin beta chain	54	5283	109	5.28%	Phycobiliprotein/Antioxidant
A0A5P8VWZ5	Glutamine synthetase	53	3812	91	4.40%	Metabolic/Nitrogen metabolism
A0A5P8W6X0	Superoxide dismutase	66	3377	84	4.07%	Antioxidant enzyme
A0A5P8W6Q0	ApcA, allophycocyanin alpha subunit	82	3106	81	3.92%	Phycobiliprotein/Antioxidant
A0A5P8VZ83	Mn-containing catalase	74	3235	79	3.82%	Antioxidant enzyme
A0A5P8VQS1	PRDX2-4, peroxiredoxin	75	3100	76	3.68%	Antioxidant/redox regulation
A0A5P8VRV8	Dps, starvation-inducible DNA-binding protein	70	2668	63	3.05%	Stress-response/genome protection
A0A5P8W4L0	ApcB, allophycocyanin beta subunit	75	2553	59	2.86%	Phycobiliprotein/Antioxidant
A0A5P8WER0	CpeA, phycoerythrin alpha chain	66	2699	57	2.76%	Phycobiliprotein/Antioxidant
A0A5P8W8N0	RNA-binding protein	51	2826	54	2.61%	Stress Adaptation
A0A5P8W4V6	DNA nickase	33	1777	45	2.18%	Stress-response/DNA maintenance
A0A5P8W0K3	CpcC, phycocyanin-associated rod linker protein	56	1734	43	2.08%	Structural/Phycobilisome stability
A0A5P8VYF8	Histidine kinase	28	1389	37	1.79%	Signal sensing/adaptation
A0A5P8W3V7	Gfo/Idh/MocA family oxidoreductase	30	1271	30	1.45%	Redox metabolism
A0A5P8W8S1	Phosphate-binding protein	18	1307	28	1.36%	Nutrient binding/metabolism
A0A5P8W131	Dps, starvation-inducible DNA-binding protein	53	1183	27	1.31%	Stress-response
A0A5P8WCP6	CpeB, phycoerythrin beta chain	39	1233	27	1.31%	Phycobiliprotein/Antioxidant
A0A5P8W3L1	Ferredoxin--NADP reductase	33	1093	26	1.26%	Redox metabolism
A0A5P8VYH0	CpcG, phycobilisome rod-core linker protein	45	803	25	1.21%	Structural/energy transfer
A0A5P8VV17	6-phosphogluconate dehydrogenase, decarboxylating	38	1093	25	1.21%	Energy metabolism
A0A5P8W3G1	ATP synthase subunit beta	41	1074	24	1.16%	Energy metabolism
A0A5P8W287	Uncharacterized protein	36	1063	23	1.11%	Unknown/putative function
A0A5P8VXU4	Inorganic pyrophosphatase	46	877	22	1.06%	Energy metabolism
A0A5P8VSL4	Red carotenoid-binding protein	34	915	21	1.02%	Antioxidant-associated

## Data Availability

All data generated or analyzed during this study are included in this published article. The mass spectrometry proteomics data have been deposited to the ProteomeXchange Consortium under the dataset identifier PXD076497. Reviewer access details can be provided upon request.
